# Postnatal Developmental Expression Profile Classifies the Indusium Griseum as a Distinct Subfield of the Hippocampal Formation

**DOI:** 10.3389/fcell.2020.615571

**Published:** 2021-01-12

**Authors:** Marie Sanders, Elisabeth Petrasch-Parwez, Hans-Werner Habbes, Monika v. Düring, Eckart Förster

**Affiliations:** Department of Neuroanatomy and Molecular Brain Research, Ruhr-University Bochum, Bochum, Germany

**Keywords:** indusium griseum, fasciola cinerea, dentate gyrus, CA2 region, Necab2, Prox1, secretagogin, PCP4

## Abstract

The indusium griseum (IG) is a cortical structure overlying the corpus callosum along its anterior–posterior extent. It has been classified either as a vestige of the hippocampus or as an extension of the dentate gyrus via the fasciola cinerea, but its attribution to a specific hippocampal subregion is still under debate. To specify the identity of IG neurons more precisely, we investigated the spatiotemporal expression of calbindin, secretagogin, Necab2, PCP4, and Prox1 in the postnatal mouse IG, fasciola cinerea, and hippocampus. We identified the calcium-binding protein Necab2 as a first reliable marker for the IG and fasciola cinerea throughout postnatal development into adulthood. In contrast, calbindin, secretagogin, and PCP4 were expressed each with a different individual time course during maturation, and at no time point, IG or fasciola cinerea principal neurons expressed Prox1, a transcription factor known to define dentate granule cell fate. Concordantly, in a transgenic mouse line expressing enhanced green fluorescent protein (eGFP) in dentate granule cells, neurons of IG and fasciola cinerea were eGFP-negative. Our findings preclude that IG neurons represent dentate granule cells, as earlier hypothesized, and strongly support the view that the IG is an own hippocampal subfield composed of a distinct neuronal population.

## Introduction

The indusium griseum (IG) is a thin bilateral stripe-like allocortical brain area covering the entire anterior–posterior extent of the corpus callosum at the basis of the cingulate cortex. According to its topographical localization, the IG can be subdivided into an anterior part bending around the genu of the corpus callosum, a dorsal part overlying the corpus callosum surface, and a posterior part around the splenium of the corpus callosum, closely associated with the fasciola cinerea (FC) (Künzle, 2004). The long anterior–posterior expansion of this otherwise narrow structure implies proximity to several different brain areas. While there is consensus to consider the IG as part of the limbic system (Di Ieva et al., 2015), its allocation to a specific neuroanatomical structure is still under debate.

The IG was initially described as a rudimental continuation of the hippocampus remaining on the dorsal surface during relocation of the hippocampus to a more basal position during phylogenesis (Elliot Smith, 1897), a view that is still favored in more recent studies (Wyss and Sripanidkulchai, 1983; Adamek et al., 1984). In more detail, the IG has been considered either as a remnant of the subiculum, of the cornu ammonis or, in particular, of the dentate gyrus (DG) connected to it via the FC. The latter, most common view is mainly based on similarities between IG and DG concerning cytoarchitecture, cell morphology, and neuronal projections (Wyss and Sripanidkulchai, 1983; Adamek et al., 1984; Laplante et al., 2013). Finally, it has also been suggested that the IG should be considered as a distinct subregion within the hippocampal formation (Künzle, 2004). The idea that the IG is more than a mere hippocampal rudiment is supported by a recent histological and magnetic resonance imaging study on the human IG clearly confirming that it does not show any signs of regression during postnatal development (Bobić Rasonja et al., 2019). Of note, the IG has gained considerable attention because astrocytes derived from this region seem to play a key role as guidepost cells for the first pioneer fibers of the murine corpus callosum (Shu et al., 2003; Smith et al., 2006; Lavado et al., 2014; Di Ieva et al., 2015). Furthermore, IG neurons in the mouse brain expressing the transcription factor Rfx3 contribute to the formation of both the hippocampal fissure and the corpus callosum (Benadiba et al., 2012). Apart from this transient function as a source of guideposts, another role of the IG during development gets significant support by a recent study describing the IG as an important prenatal relay station in developing limbic and olfactory circuits (Fuzik et al., 2019). However, more specific functions of the mature IG remain elusive.

To clarify the ongoing controversial discussion on the identity of the IG, we analyzed the postnatal spatiotemporal expression pattern of calbindin, secretagogin, N-terminal EF-hand calcium-binding protein 2 (Necab2), and of calmodulin-binding Purkinje cell protein 4 (PCP4) in the mouse IG, FC, and hippocampus. Finally, the previously hypothesized common identity of IG pyramidal and dentate granule cells was explored by investigating the protein expression of the transcription factor Prospero-related homeobox protein 1 (Prox1), known to define dentate granule cell fate, and by analyzing IG, FC, and DG neurons in transgenic mice that express enhanced green fluorescent protein (eGFP) in early postmitotic dentate granule cells (Overstreet et al., 2004).

## Materials and Methods

### Animals

All mice were treated in accordance with the guidelines of the German animal protection law, maintained in a dark–light cycle of 12/12 h, and provided with food and water *ad libitum*. C57BL/6J wild-type mice were purchased from the Jackson Laboratory (Bar Harbor, ME, United States) and bred in the animal house of the Medical Faculty, Ruhr-University Bochum (Germany).

To cover broad developmental stages, we used two mice each at postnatal day (p) 0, 5, 15, 20, 25, and 6 months of age for cresyl violet staining, as well as two mice each at p 0, 2, 5, 8, 10, 12, 15, 20, and 25, and two adult mice each, aged 6 and 12 months for immunostaining.

To compare immature dentate granule cells with IG and FC neurons, brains of proopiomelanocortin (POMC)–eGFP transgenic mice (Overstreet et al., 2004), bred in the animal facility of the Center for Molecular Neurobiology Hamburg (ZMNH, University Medical Center Hamburg-Eppendorf, Germany), were examined at p0.

### Tissue Preparation

Mice aged p0–p25 were deeply anesthetized by CO_2_ asphyxiation and decapitated. The brains were quickly removed, immersion fixed in 4% paraformaldehyde (PFA) in 0.1 M phosphate buffer (pH 7.4) for 1 week, and then transferred to 0.5% PFA until further processed. Adult mice aged 6 and 12 months were deeply anesthetized with pentobarbital potassium (Narcoren^®^, 500 mg/kg, Merial GmbH, Germany) as described (Petrasch-Parwez et al., 2007). Briefly, after opening the thorax, 0.3 mL Liquemin N 25000 (Roche, Basel, Switzerland) was intracardially injected followed by a transcardial perfusion with 4% PFA for 20 min. The brains were removed, post-fixed overnight in 4% PFA, and then transferred to 0.5% PFA in 0.1 M phosphate buffer (pH 7.4) until used.

For peroxidase immunohistochemistry, all brains were cut into 40-μm-thick coronal vibratome sections starting anterior of the corpus callosum genu, ending occipital of the corpus callosum splenium and immunostained free floating as described below.

For immunofluorescence histochemistry, POMC-eGFP transgenic mice were decapitated at p0. Entire brains were immersion-fixed in 4% PFA for 1 week, washed in 0.1 M phosphate buffer, cut into 50-μm vibratome sections, mounted on Superfrost Slides, air-dried for 30 min, and subsequently stained as described below.

### Peroxidase Immunohistochemistry

Sections were immunostained with primary antibodies in a repeated order as previously described (Petrasch-Parwez et al., 2007). Every 10th section of the brains was stained with primary antibodies against calbindin (Swant, Marly, Switzerland; Cat# CB 38, RRID: AB10000340; dilution: 1:20,000), secretagogin (Sigma–Aldrich, Munich, Germany; Cat# HPA006641, RRID: AB1079874; dilution: 1:2,000), Necab2 (Sigma–Aldrich, Munich, Germany; Cat# HPA013998, RRID: AB1848016; dilution: 1:20,000), PCP4 (Sigma–Aldrich, Munich, Germany; Cat# HPA005792, RRID: AB1855086; dilution: 1:5,000), and Prox1 (Millipore, Billerica, United States; Cat# AB5475, RRID: AB177485; dilution: 1:5,000).

Peroxidase immunohistochemistry was performed as previously described (Petrasch-Parwez et al., 2007). Briefly, the sections were reduced in 1% NaBH_4_ in phosphate-buffered saline (PBS) for 30 min, blocked in 10% normal goat serum (NGS, Interchem, Pfaffen-Schwabenheim, Germany) in PBS with 0.3% Triton X-100 (SERVA Electrophoresis GmbH, Heidelberg, Germany), and then incubated with the respective primary antibody diluted in the blocking solution for 72 h at 4°C. Afterward, the sections were rinsed in PBS, pre-incubated in 0.1% bovine serum albumin (BSA, 11930, SERVA Electrophoresis GmbH, Heidelberg, Germany) in PBS for 30 min, and incubated with a biotinylated goat anti-rabbit antibody (Vector Laboratories, Burlingame, CA, United States) overnight at 4°C. Next day, the sections were again rinsed in PBS, incubated in 0.1% BSA in PBS, and treated with an avidin-biotinylated peroxidase complex (Vector Laboratories) for 4 h at room temperature. Peroxidase was visualized with 3,3’-diaminobenzidine. Finally, all sections were mounted on Superfrost Plus slides, air-dried for an hour, quickly dehydrated, and coverslipped.

### Cresyl Violet Staining

Every 10th section was stained with 0.5% cresyl violet solution (pH 4.8, Certistain^®^, Art. Nr. 1.05235, Merck KGaA, Darmstadt, Germany) for morphological reference of adjacent immunostained sections.

Photodocumentation of cresyl violet and peroxidase-immunostained sections was performed with an Olympus Microscope BH-2 equipped with a camera Olympus DP-71 (Olympus, Japan) and the computer-assisted software analysis Cell A (Soft imaging system GmbH, Germany). The images were photoprocessed as TIFF files using Adobe Photoshop (version 2015) to adjust contrast and brightness.

### Immunofluorescence Histochemistry

The mounted slides were rinsed twice in PBS, preincubated in 10% NGS for 30 min, and incubated with the antibody against Necab2 (1:1,000), diluted in the previous solution for 24 h. Then, slices were washed in PBS, preincubated with a 0.2% BSA/PBS solution for 30 min, and incubated with a red-fluorescently labeled secondary antibody (Alexa Fluor^®^ 633, Thermo Fisher Scientific Inc., Waltham, United States; RRID: AB2535732; 1:1,000) in 0.2% BSA/PBS solution. After final washing in PBS, the slides were coverslipped in anti-fade medium (Prolog Gold, Molecular Probes^®^, Thermo Fisher Scientific Inc., Waltham, United States). The immunofluorescence staining was imaged with an inverted Confocal Spinning-Disc-Laser Microscope (VisiScopeConfocal—Cell Explorer, Visitron Systems GmbH, Puchheim, Germany) and the computer-assisted software cellSens Entry (Olympus Life Science Solutions, Hamburg, Germany) and photoprocessed as described above.

## Results

### Cytoarchitecture of the IG and FC

**FIGURE 1:**
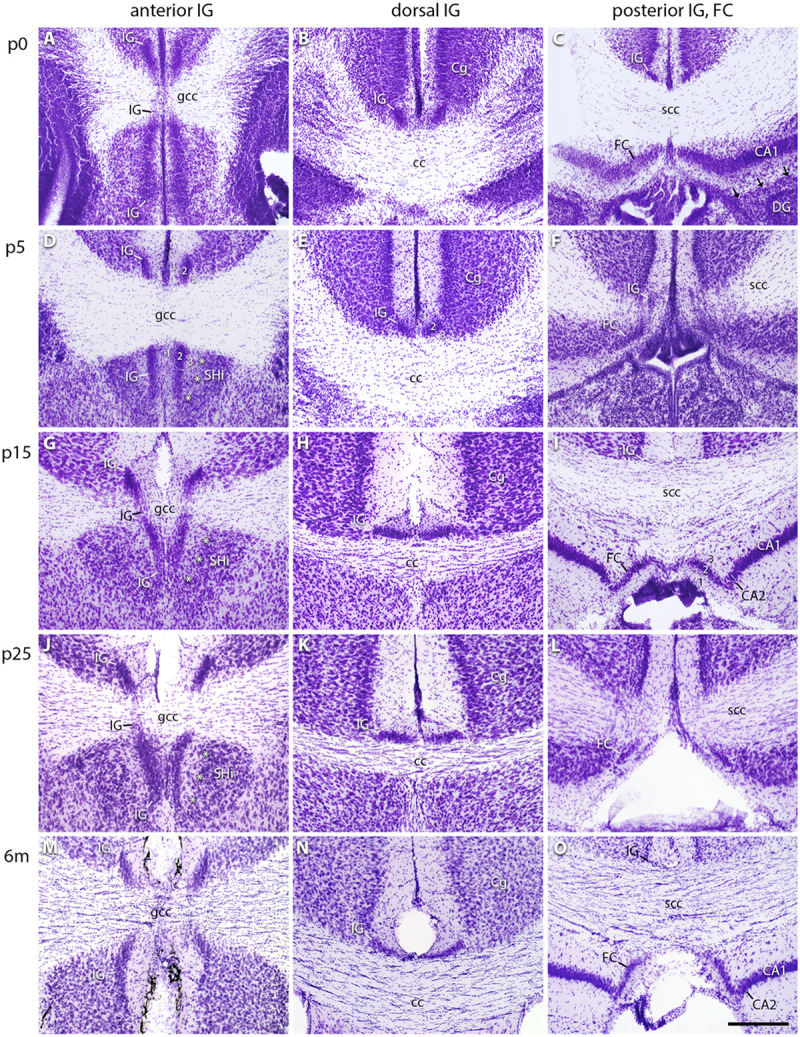
Cytoarchitecture of the indusium griseum (IG) subdivisions. Cresyl violet–stained coronal sections of IG and FC in the mouse brain at p0 **(A–C)**, p5 **(D–F)**, p15 **(G–I)**, p25 **(J–L)**, and 6 months (m) of age **(M–O)** showing the anterior **(A,D,G,J,M)**, dorsal **(B,E,H,K,N)**, and posterior IG, the latter closely associated with the FC **(C,F,I,L,O)**. Layers of the IG (1, 2, 3 in **D,E,I**) differentiate during maturation, i.e., the IGs pyramidal cell layer (2, in **D,E**) flattens, and the polymorph layer **(D,E)** narrows with maturation, while the morphological adaptation of the molecular layer is less remarkable **(B,E,H,K,N)**. Note the bending of the IG around the genu of corpus callosum (gcc, in **A,G,J,M**) and the transition of IG and FC around the splenium of corpus callosum (scc, in **F,L**). The IG is neighboring the cingulate cortex (Cg) and, in its most anterior part, the septohippocampal nucleus (SHi). Asterisks show the border between IG and SHi (*** in **D,G,J**). The FC is localized adjacent to the cornu ammonis (CA)1 **(C)** or to the medial CA2 region **(I,O)**, but separated from the dentate gyrus (DG) through the hippocampal fissure (arrows in **C**). Differences in section level are due to tilted angles of section series. Scale bar = 200 μm in **(O)** for **(A–O)**.

Cresyl violet staining of serial brain sections was performed to determine the spatial extension of the IG and FC subdivisions during development (Figure 1). These sections were also used as a morphological reference for immunostained sections. The IG and FC were identified according to the mouse brain atlases by Paxinos and Watson (2009) and Paxinos and Franklin (2019). At all postnatal stages, the subdivision of the IG into an anterior (Figures 1A,D,G,J,M), dorsal (Figures 1B,E,H,K,N), and posterior part was confirmed (Figures 1C,F,I,L,O). The demarcation of the IG from the overlying cingulate cortex was already visible at p0 (Figures 1A,B). We detected a transition zone between IG and FC (Figure 1F), not observed between the IG and DG, which were always separated through the hippocampal fissure (Figure 1C). Of note, the FC directly neighbors the CA1 region, the medial part of CA2, or—more posteriorly—the subiculum (Figures 1C,F,I,L,O). The FC can be recognized by its pyramidal cell layer, which is oriented perpendicular to the hippocampal pyramidal cell layer (Figures 1I,O). The neuronal cell density of the IG decreased from anterior to posterior part in adult mice (Figures 1G–I,M–O). While the anterior IG is clearly separated by the fissura longitudinalis cerebri (Figures 1A,D,G,J,M), the dorsal IG partly merges with its contralateral counterpart (Figures 1B,E,H,K,N). The posterior IG is again divided into two separate parts by the fissura longitudinalis cerebri (Figures 1C,F).

### Calbindin Expression Decreases During Postnatal IG and FC Development

**FIGURE 2:**
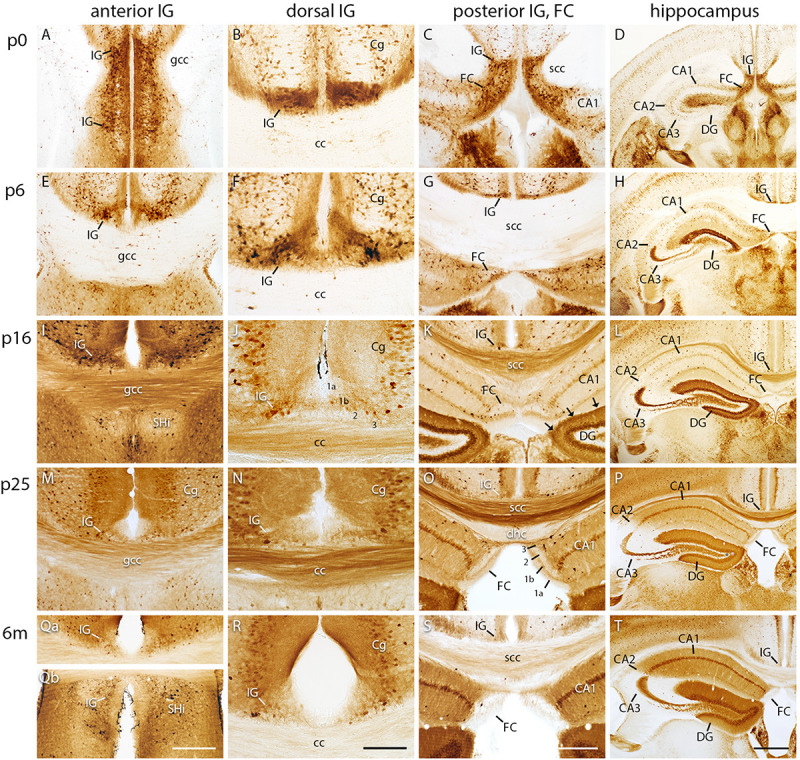
Calbindin immunohistochemistry in indusium griseum (IG), fasciola cinerea (FC), and hippocampus. Coronal sections of mouse brain at p0 **(A–D)**, p5 **(E–H)**, p15 **(I–L)**, p25 **(M–P)**, and 6 months (m) of age **(Qa–T)** show the anterior **(A,E,I,M,Qa,Qb)**, dorsal **(B,F,J,N,R)**, and posterior IG, FC **(C,G,K,O,S)**, and hippocampus **(D,H,L,P,T)**. **(Qa,Qb)** show the same section. Note the postnatal decrease of calbindin reactivity in IG and FC with calbindin-negative neurons in the pyramidal cell layer **(A,E,F,J, K–T)**. **(A)** shows the IG bending around the genu of corpus callosum (gcc). **(C,D)** display the transition of IG and FC around the splenium of corpus callosum (scc). Layers are exemplarily numbered (1a, 1b, 2, 3) in J for IG and in **(O)** for FC. The outer molecular layer is faintly stained (1a) compared to the moderately stained inner molecular layer (1b) in IG and FC. The pyramidal cell layer (2 in **J,O**) of the IG and FC contains both intensely and faintly stained positive cells **(E,F,I–K,M–O,Qa–T)**. Cornu ammonis (CA) 2 and 3 show a developmentally decreasing reactivity, except for the strong staining of stratum lucidum. In contrast, CA1 and dentate gyrus (DG) show a developmentally increasing reactivity, reflecting their characteristic lamination **(D,H,L,P,T)**. The hippocampal fissure (arrows in **K**) clearly delimits the FC from the DG **(C,K,O,S)**. Cg, cingulate cortex. 3 in **(J,O)** polymorph layer of IG and FC. Scale bar = 200 μm in **(Qb)** for **(A,E,I,M,Qa,Qb)**; scale bar = 100 μm in **(R)** for **(B,F,J,N,R)**; scale bar = 100 μm in S for **(C,G,K,O,S)**; and scale bar = 400 μm in **(T)** for **(D,H,L,P)** and **(T)**.

Calbindin is known as a marker to distinguish hippocampal subdivisions (Celio, 1990; Kjonigsen et al., 2011). Therefore, we investigated its spatiotemporal distribution in the IG and FC compared to the hippocampus and DG (Figure 2). At p0, calbindin was strongly expressed in the anterior, dorsal, and posterior part of the IG and in the FC (Figures 2A–C). Calbindin-positive neurons accumulated particularly adjacent to IG pyramidal cells, which mainly lacked calbindin staining (Figures 2A,B,F,J,M,N). During postnatal development, calbindin expression gradually decreased in all subdivisions (Figures 2A–C,E–G,I–K,M–O). In the adult IG, only a few calbindin-positive neurons were detected within a faintly stained neuropil (Figures 2Qa–S). In detail, at p0, the IG contained numerous, intensely immunostained neurons at the border between the predominantly immunonegative IG pyramidal cell layer and the IG molecular layer, and few positive neurons that were scattered in the molecular and polymorph layer. At p5, calbindin-positive neurons were distributed in all three layers (Figure 2F) and were closely neighboring immunonegative pyramidal cells (Figures 2E,F). From stage p10 on, the density of calbindin-positive cells in the IG continuously decreased to few scattered neurons from p15 on into adulthood (Figures 2J,N,R). Beaded axons of calbindin-positive cells projected to the adjacent cortical areas, the corpus callosum, and to the contralateral hemisphere (Supplementary Figure S1A). As already detected in the IG, the FC also contained a large number of calbindin-immunopositive neurons at p0 (Figure 2C), which subsequently decreased during postnatal development (Figures 2G,K,O,S). Notably, calbindin immunostaining at p0 confirmed the conjunction of IG and FC (Figures 2C,D) as shown by cresyl violet staining. In the hippocampus proper and in the DG, calbindin immunostaining increased during postnatal development (Figures 2D,H,L,P,T). While positive cells were mainly found in the outer dentate granule cell layer during the first postnatal weeks, the subgranular zone of the DG in contrast, known to harbor neuronal stem cells, was immunonegative (Figures 2D,H,L,P). Almost all dentate granule cells were calbindin-positive in the adult mice (Figure 2T). Altogether, our investigations show that calbindin is a suitable marker for the IG and FC during early postnatal development, but not in adult mice, much in contrast to the calbindin expression in the hippocampus proper and DG.

### Secretagogin Emerges Late During Postnatal IG and FC Development

**FIGURE 3:**
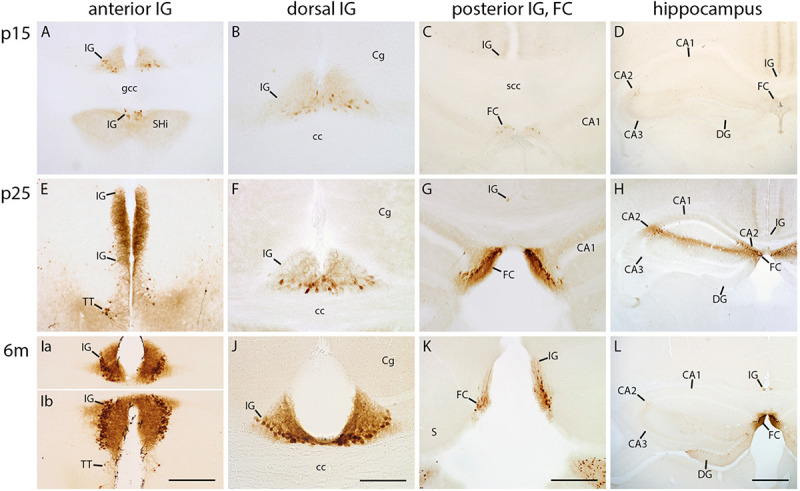
Secretagogin immunohistochemistry in indusium griseum (IG), fasciola cinerea (FC) and hippocampus. Coronal sections of mouse brain at p15 **(A–D)**, p25 **(E–H)**, and 6 months (m) of age **(Ia–L)** show the anterior **(A,E,Ia,Ib)**, dorsal **(B,F,J)** and posterior IG, the FC **(C,G,K)** and hippocampus **(D,H,L)**. **(Ia,Ib)** belong to the same section. **(E)** displays the molecular layer of the most anterior IG part bending around the genu of corpus callosum (gcc); **(K)** exhibits the transition of IG and FC around the splenium of corpus callosum (scc). Because of tilted angle of the sections, the anterior IG is adjacent to, but delimited from, the septohippocampal nucleus (SHi) and tenia tecta (TT in **A,E,Ib**). IG and FC show a postnatally increasing secretagogin reactivity most prominent in the adult anterior **(Ia,Ib)** and dorsal IG **(J)**. Note that in the adult IG, as earlier in development, only a subpopulation of neurons in the pyramidal cell layer is secretagogin-positive **(Ia,Ib,J)**. The cornu ammonis (CA) 2 is only moderately stained at P25 **(H)**. In the dentate gyrus (DG) very few faintly stained neurons are localized at the border between the molecular and granule cell layer **(C,D,G,H,K,L)**. Secretagogin-staining clearly delimits the dorsal IG from the cingulate cortex (Cg), most obvious in adult mice **(J)**. Scale bar = 200 μm in **(Ib,K)** for **(A,C,E,G,Ia,Ib,K)**; scale bar = 100 μm in **(J)** for **(B,F,J)**; scale bar = 400 μm in **(L)** for **(D,H,L)**.

Secretagogin has previously been described as a marker for the IG in adult mice (Mulder et al., 2009). Therefore, we wanted to explore its developmental expression in the IG and FC. At p0, both the IG and FC lacked secretagogin reactivity (data not shown). At p5, only few faintly stained secretagogin-positive neurons were detected in the IG. Their quantity and staining intensity started to increase from the second postnatal week on, progressing from anterior to posterior (Figures 3A–C). In the anterior IG, positive cells were predominantly densely arranged within the pyramidal cell layer (Figures 3A,E,Ia,Ib), whereas the dorsal part showed many immunopositive, but also unstained cells (Figures 3B,F,J). The majority of secretagogin-positive dendrites were oriented towards the molecular layer; some dendrites traversed to the contralateral IG (Supplementary Figure S1B). The heterogeneity, laminar distribution, and developmental expression pattern of secretagogin-positive cells in the FC were similar to those of the anterior IG (Figures 3C,G,K). The hippocampal CA2 region transiently showed a similar staining pattern as the IG from p15 to p25, with many secretagogin-positive pyramidal cells and an immunopositive stratum oriens and radiatum (Figures 3D,H). Later, in turn, the adult hippocampal CA2 region almost lacked secretagogin staining (Figure 3L). Faintly stained secretagogin-positive neurons appeared at the border of the dentate granule cell layer and the inner molecular layer in adult mice DG (Figure 3L). Our findings show that secretagogin is a useful marker for the late postnatal and mature IG and FC, but not for the early postnatal IG and FC. Finally, secretagogin expression of the hippocampal CA2 region was restricted to a limited time window during postnatal development.

### Necab2 Is a Novel Marker for IG and FC

**FIGURE 4:**
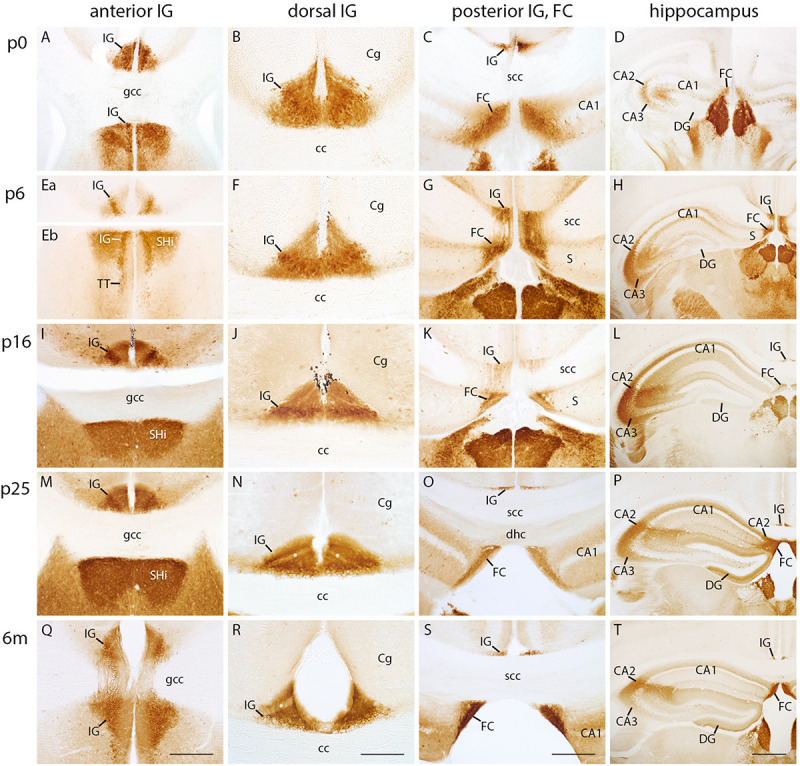
Necab2 immunohistochemistry in indusium griseum (IG), fasciola cinerea (FC), and hippocampus. Coronal sections of mouse brain at p0 **(A–D)**, p5 **(Ea–H)**, p15 **(I–L)**, p25 **(M–P)**, and at 6 months (m) of age **(Q–T)** showing the anterior **(A,Ea,Eb,I,M,Q)**, dorsal **(B,F,J,N,R)**, and posterior IG, FC **(C,G,K,O,S)**, and hippocampus **(D,H,L,P,T)**. **(Ea,Eb)** belong to the same section. IG, FC, and cornu ammonis (CA) 2 display strong Necab2 immunostaining at all postnatal stages. **(Q)** exhibits the anterior IG bending around the genu of corpus callosum (gcc). IG and FC merge at the splenium of corpus callosum (scc) **(G,K)**. The anterior IG is localized close to the septohippocampal nucleus (SHi) and tenia tecta (TT) **(A,Eb,Q)**; the FC is adjoining the medial CA2 subfield **(P)**. Note the postnatal differentiation of IG layers **(B,F,J,N,R)**. CA1 pyramidal and granule cell layer of dentate gyrus (DG) lack Necab2 reactivity **(D,H,L,P,T)**. Necab2 clearly delimits the IG from overlying cingulate cortex (Cg) **(B,F,J,N,R)** and the FC from adjacent subiculum (S) **(G,H,K)**. Scale bar = 200 μm in **(Q)** and S for **(A,C,Ea,Eb,G,I,K,M,O,Q,S)**; scale bar = 100 μm in **(R)** for **(B,F,J,N,R)**; scale bar = 400 μm in **(T)** for **(D,H,L,P,T)**.

Necab2 has been shown to be a valuable marker for hippocampal subdivisions (Zimmermann et al., 2013). We therefore, investigated the developmental Necab2 protein expression in the IG and adjacent structures. Interestingly, Necab2 immunostaining in the IG and FC was already prominent at p0 (Figures 4A–C). The intense immunostaining was consistent through all postnatal stages along the entire anterior–posterior extent of the IG and FC (Figure 4), thereby designating Necab2 as a novel marker for these structures. In more detail, both, IG and FC, revealed a trilaminar staining pattern with a strong immunoreactivity in all layers. The pyramidal cell layer exhibited many immunopositive and scattered immunonegative neurons (Figures 4A–C,E–G,I–K,M–O,Q–S). Necab2 staining also confirmed the conjunction between IG and FC (Figure 4G). Importantly, the dentate granule cell layer was devoid of Necab2 staining at all investigated stages, whereas the hippocampal CA2 region displayed Necab2-staining throughout postnatal development into adulthood, although slightly delayed (starting by p5) when compared to the IG (Figures 4D,H,L,P,T). In conclusion, we show that Necab2 is a reliable marker for the IG and FC throughout all postnatal stages, in contrast to the immunonegative dentate granule cell layer, which was devoid of Necab2.

### PCP4 Expression Decreases in the IG During Postnatal Development

**FIGURE 5:**
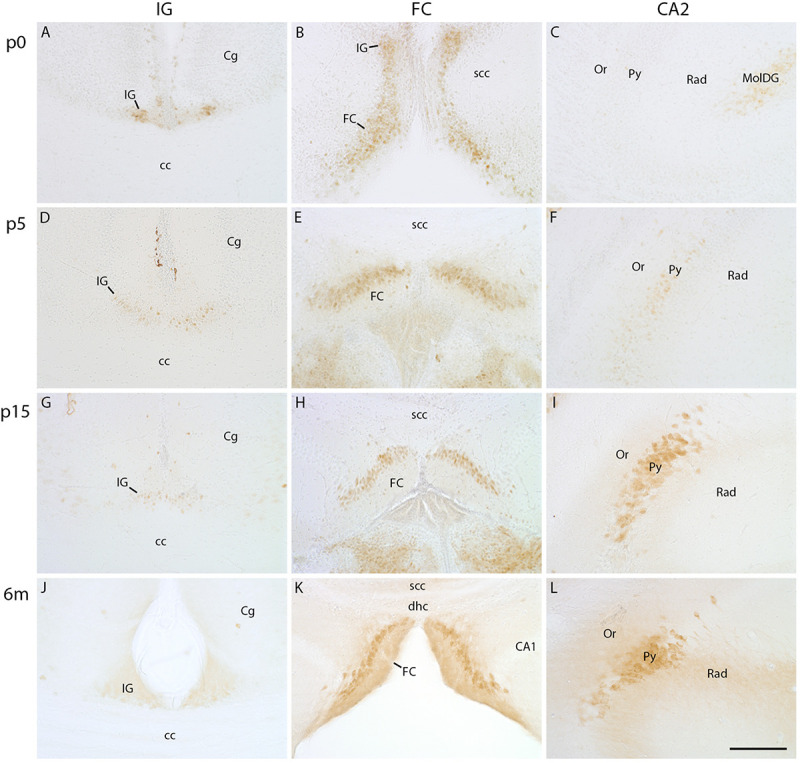
PCP4 immunohistochemistry in indusium griseum (IG), fasciola cinerea (FC), and hippocampus. Coronal sections of mouse brain at p0 **(A–C)**, p5 **(D–F)**, p15 **(G–I)**, and at 6 months (m) of age **(J–L)** show faint staining of PCP4 in IG at p0 further decreases with age **(A,D,G,J)**, whereas staining of FC **(B,E,H,K)** and CA2 **(C,F,I,L)** increases with age **(C,F,I,K)**. cc, corpus callosum; Cg, cingulate cortex; dhc, dorsal hippocampal continuation; MolDG, molecular layer of dentate gyrus; Or, stratum oriens; Py, pyramidal cell layer; Rad, stratum radiatum; scc, splenium of corpus callosum. Scale bar in **(L)** = 100 μm for **(A–L)**.

As Necab2 and secretagogin immunostaining pointed to similarities between IG, FC, and CA2, we studied the expression of PCP4, a specific marker for CA2 pyramidal cells (Lein et al., 2005; Laeremans et al., 2013; San Antonio et al., 2014). At p0, faint PCP4 immunostaining was visible in the IG and FC with a continuous transition of PCP4-positive cells from IG to FC (Figure 5B), whereas CA2 lacked PCP4 at that stage (Figure 5C). During postnatal development, the intensity of initially PCP4-positive IG cells (Figure 5A) declined, and later the IG was devoid of PCP4 immunoreactivity (Figures 5D,G,J). In contrast, the pyramidal cell layer of the FC (Figures 5B,E,H,K) contained PCP4-positive neurons at all postnatal stages (Figures 5B,E,H,K) and the CA2 region from around p5 on (Figures 5C,F,I,L), both with increasing intensity. Altogether, PCP4 immunostaining revealed characteristic spatiotemporal differences in the investigated subregions, i.e., a developmental decrease of IG and an increase of PCP4-positive FC and CA2 neurons.

### IG and FC Neurons Do Not Express the Dentate Granule Cell Marker Prox1

**FIGURE 6:**
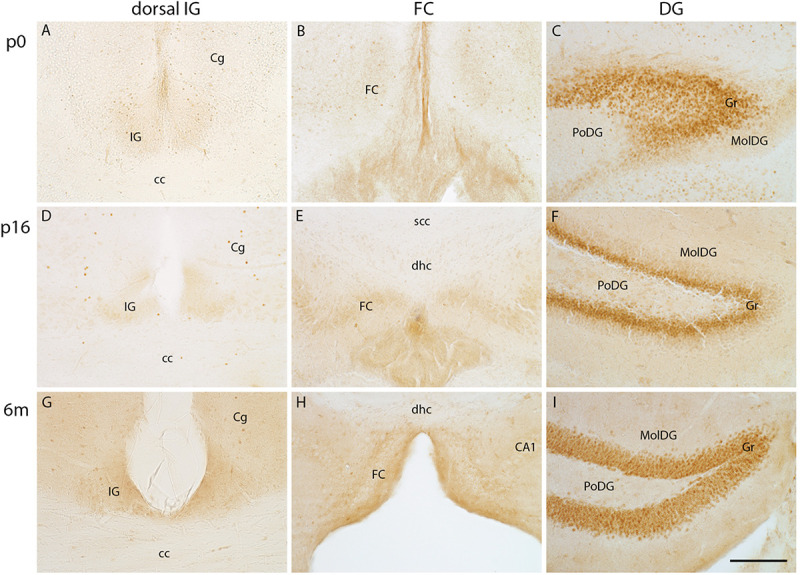
Prox1 immunohistochemistry in indusium griseum (IG), fasciola cinerea (FC), and dentate gyrus (DG). Coronal vibratome sections of IG **(A,D,G)** and FC **(B,E,H)** lack Prox1-immunopositive pyramidal cells, whereas the dentate granule cell layer (Gr) is intensely positive for Prox1 from p0 to adulthood **(C,F,I)**. cc, corpus callosum, Cg, cingulate cortex; dhc, dorsal hippocampal continuation; MolDG, molecular layer of DG; PoDG, polymorph layer of DG; scc, splenium of corpus callosum; Scale bar in **(I)** = 100 μm for **(A–I)**.

Previous studies described a similar morphology of IG pyramidal and dentate granule cells (Adamek et al., 1984). Prox1, a transcription factor that is expressed in dentate granule cells throughout embryonic and postnatal development into adulthood, is known to define dentate granule cell fate (Lavado and Oliver, 2007; Iwano et al., 2012). Here, we investigated Prox1 protein expression in the IG and FC from early postnatal stages to adulthood. The Prox1 antibody immunostained nuclei of dentate granule cells at all postnatal stages (Figures 6C,F,I), most intensely in the subgranular zone (Figure 6F) adjacent to the polymorph layer of the DG. In contrast, Prox1 immunostaining was absent in the IG (Figures 6A,D,G) and FC (Figures 6B,E,H) at all stages investigated. In conclusion, Prox1 immunostaining clearly distinguished IG and FC neurons from dentate granule cells.

### eGFP Expression in POMC-eGFP Mice Discerns IG and FC Neurons From Dentate Granule Cells

**FIGURE 7:**
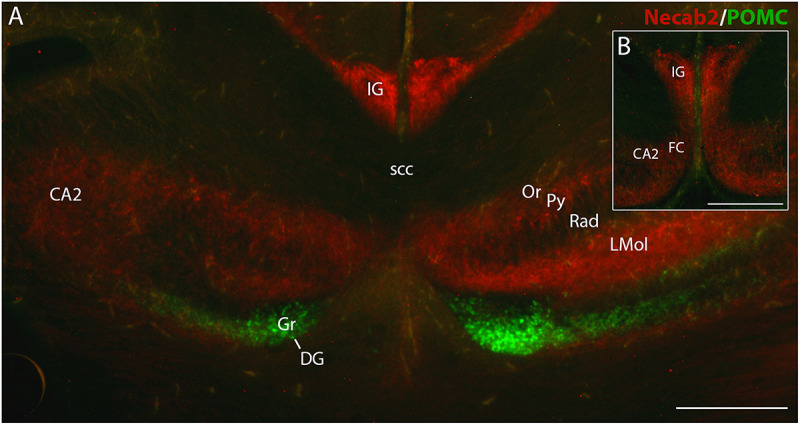
Necab2 immunofluorescence in indusium griseum (IG), fasciola cinerea (FC), and hippocampus in a POMC-eGFP transgenic mouse brain at p0. Adjacent coronal sections show non-overlapping expression of POMC-eGFP (green) and Necab2 immunofluorescence (red). POMC-eGFP is localized in granule cells (Gr) of dentate gyrus (DG). IG, FC, and cornu ammonis (CA) are Necab2-immunopositive **(A,B).** Note the close topographical association of IG, FC, and medial CA2 **(B)**. Necab2 appears more intense in IG than in CA2 and FC **(B)**. It is also detected in the lacunosum molecular layer (LMol) of CA, in the stratum oriens (Or) and pyramidal cell layer (Py) of CA2. CA1 shows faint immunofluorescence in Or, Py, and stratum radiatum (Rad). scc, splenium of corpus callosum. Scale bar in **(B)** = 20 μm for **(A,B)**.

Finally, we analyzed brain sections of p0 transgenic mice expressing eGFP under control of POMC promotor sequences. In this mutant, newly generated postmitotic dentate granule cells transiently express eGFP for approximately 4 weeks (Overstreet et al., 2004). Fluorescence microscopy confirmed that dentate granule cells expressed eGFP. In contrast, in the IG, the FC and the hippocampal pyramidal cell layer eGFP expression was absent (Figure 7). *Vice versa*, fluorescent immunostaining with the Necab2 antibody labeled IG and FC neurons, but not dentate granule cells (Figures 7A,B). Necab2 immunofluorescence also confirmed that IG and FC form a continuum toward the hippocampal CA2 region (Figure 7B). Thus, combined eGFP fluorescence and Necab2 immunofluorescence clearly distinguished IG and FC neurons from dentate granule cells.

## Discussion

In the present study, we characterized the developmental expression pattern of the calcium-binding proteins calbindin, secretagogin, and Necab2, the calmodulin-binding protein PCP4, the transcription factor Prox1, and of eGFP under control of the POMC promotor in transgenic mice, in the IG, FC, and the hippocampus. We found that Necab2 is strongly expressed in the IG and FC during all postnatal stages until adulthood, identifying Necab2 as a novel reliable marker for the mouse IG and FC. In addition, the IG forms a continuous Necab2-immunopositive structure together with the FC, closely neighboring the medial portion of the hippocampal CA2 region. In contrast, calbindin, secretagogin, and PCP4 are only transiently expressed in the IG and FC.

Remarkably, IG neurons did not express Prox1, a transcription factor known to define granule cell fate, thereby disproving the often-stated hypothesis that IG neurons have the same developmental fate as dentate granule cells. Moreover, our finding that IG and FC neurons, much in contrast to dentate granule cells, did not express eGFP under control of the POMC promotor in a transgenic mouse model corroborates this interpretation. Our immmunohistochemical data on calbindin, Necab2, and Prox1 are bolstered by *in situ* hybridization data for the RNA encoding these proteins (© 2015 Allen Institute for Brain Science. Allen Brain Atlas API. Available from: Calbindin: https://mouse.brain-map.org/experiment/thumbnails/79556672?image_type=ish&popup=true; Necab2: http://developingmouse.brain-map.org/experiment/thumbnails/73788010?image_type=ish&popup=true; Prox1: http://mouse.brain-map.org/experiment/thumbnails/73520980?image_type=ish&popup=true). Altogether, our findings strongly support the view that the IG should be addressed as a distinct subregion of the hippocampal formation.

### Calcium- and Calmodulin-Binding Proteins Characterize IG and FC

Calcium-binding proteins are important for the regulation of intracellular calcium homeostasis and transport. The here examined expression patterns of the calcium-binding proteins calbindin, secretagogin, and Necab2 have been described as mostly non-overlapping, making them useful markers to identify selective brain areas and specific neuronal subpopulations (Celio, 1990; Jacobowitz and Winsky, 1991; Mulder et al., 2009; Zimmermann et al., 2013). Mulder et al. (2009) demarcated the calbindin-positive cingulate cortex from the calbindin-negative IG. Our findings, however, reveal that calbindin is temporally strongly expressed in the IG, i.e., during early postnatal development, decreasing continuously until adulthood. In addition, the here observed spatiotemporal expression of calbindin clearly differs between IG and FC compared to the DG, as the expression of calbindin increases during maturation of dentate granule cells.

Secretagogin is expressed in neurons of the mammalian corticolimbic system (Mulder et al., 2009); immunopositive cells were shown to be positioned along the FC, the IG, and anterior hippocampal continuation. As IG neurons, i.e., secretagogin-positive neurons, morphologically and physiologically resemble dentate granule cells (Wyss and Sripanidkulchai, 1983; Adamek et al., 1984; Mulder et al., 2009), a common developmental origin of dentate granule cells and IG pyramidal cells has been proposed. This hypothesis has recently been questioned by Fuzik et al. (2019), who detected that the IG neurons lack Prox1-expression in young adult mice. This observation was confirmed in our study and even substantially extended, as we included newborn mice in our investigation on the IG and FC, both of which were also devoid of Prox1 immunoreactivity. The presence of scattered secretagogin-positive cells at the border between dentate granule cell and inner molecular layer (Mulder et al., 2009) was also confirmed in our study.

Secretagogin-positive cells are often found in vicinity to the ventricular system with dendrites oriented along the pial surface, which might display a role in releasing neuroactive substances into the cerebrospinal fluid (Mulder et al., 2009, 2010). Further assumed functions are a neuroendocrine role in vesicle exocytosis (Bauer et al., 2011), microtubules dynamics (Maj et al., 2010, 2012), and a possible neuroprotective role against the neurodegeneration as in Alzheimer disease (Attems et al., 2008). Altered secretagogin expression has been described to possibly reflect cellular dysfunction of locus coeruleus neurons in Alzheimer disease (Zahola et al., 2019). Moreover, secretagogin may be involved in neuronal differentiation persisting during neurogenesis until adulthood (Alpár et al., 2012). In this regard, it would be of great interest to further examine the function of secretagogin-positive cells within the IG and FC.

Fuzik et al. (2019) detected calbindin D28k RNA within secretagogin-positive excitatory neurons while we observed a mainly non-overlapping time course of calbindin and secretagogin protein expression within the IG and FC. Our finding that both calbindin expression and secretagogin expression within the IG and FC have a characteristic time course during postnatal development reflects a particular differentiation of IG neurons and argues against the view that the IG might represent a mere hippocampal rudiment, as stated earlier (Elliot Smith, 1897; Macchi, 1951; Di Ieva et al., 2015). Instead, the characteristic postnatal time course of calcium-binding protein expression argues in favor of a functional role of the IG beyond its transient role as a source of guidepost cells in mice, required for the formation of the murine corpus callosum (Shu et al., 2003; Smith et al., 2006; Lavado et al., 2014; Di Ieva et al., 2015). This assumption is in line not only with the results of Fuzik et al. (2019) as mentioned above, but also with recent findings revealing a continuous maturation of the fetal human IG (Bobić Rasonja et al., 2019) and clearly excluding its postnatal regression.

In our study, we identified Necab2 as a novel reliable marker protein for the anterior, dorsal, and posterior IG and also for the FC throughout all postnatal stages, clearly demarcating these brain areas from adjacent structures. The strong and selective Necab2 reactivity will facilitate future studies on the IG and FC, as these narrow structures are difficult to delimit especially in their anterior and posterior parts. It would be of great interest to investigate Necab2 in the respective brain areas of other mammalian species including humans. Necab1, Necab2, and Necab3 are evolutionary highly conserved homolog calcium-binding proteins that were originally described as targets of the calcium sensorsynaptotagmin I and of the transcription factor Pax6, which is an important regulatory gene for proper eye and brain development (Bernier et al., 2001; Sugita et al., 2002). Interestingly, Necab2 has been identified as a pronociceptive molecular determinant of inflammatory pain within dorsal root ganglia and spinal cord interneurons (Zhang et al., 2018). Its downregulation prevented persistent pain and facilitated functional recovery (Zhang et al., 2018).

PCP4 has been described as a specific marker for the hippocampal CA2 region from around P4 on, for the CA3 region, and the dentate granule cell layer (Lein et al., 2005; San Antonio et al., 2014). The medial CA2 region and the FC form a PCP4-positive continuum as we confirm in our study, even though neurons of these two regions are not identical (Lein et al., 2005). PCP4 is known to play an important role concerning neuroendocrine cell differentiation and release of neurotransmitters (Ziai et al., 1986, 1988; Harashima et al., 2011; Renelt et al., 2014) and as an inhibiting modulator of Ca^2+^–calmodulin signaling controlling the resistance of neurons to toxicity (Recabarren and Alarcón, 2017). Dysregulated PCP4 expression was described in the context of neuronal disorders such as Alzheimer disease (Slemmon et al., 1994; Recabarren and Alarcón, 2017), Huntington disease (Utal et al., 1998), major depressive disorder (Teyssier et al., 2011), and Down syndrome (Cabin et al., 1996; Chen et al., 1996) and related to alterations in the prefrontal cortex of individuals suffering from alcohol abuse (Iwamoto et al., 2004). The human IG has been reported to be more resistant to factors triggering histological changes occurring in the aged and Alzheimer diseases hippocampus, i.e., to the production of abnormal proteins associated with Alzheimer disease (Lippa et al., 1990; Lippa and Smith, 1992). Considering the fact that the CA2 region of the hippocampus also belongs to the brain regions that have been described as more resistant to degeneration and the occurrence of neurofibrillary tangles (Ishizawa et al., 2002), further studies on the apparently partially overlapping protein expression pattern of IG, FC, and CA2 could be of great interest. Notably, our findings revealed spatiotemporal differences in PCP4 expression concerning the IG, FC, and CA2. It would be interesting to expand the comparison between IG, FC, and CA2 by using additional markers that may elucidate functional differences of these areas.

### Prox1 and POMC Promoter–Driven eGFP Expression in Dentate Granule Cells

Prox1 has first been cloned and described by Oliver et al. (1993) as a mouse homolog of the *Drosophila* homeobox gene prospero, playing a role in early development of the central nervous system. It has been identified as the transcription factor that defines dentate granule cell identity, and it regulates cell proliferation in the dentate neuroepithelium during early development (Lavado et al., 2010; Iwano et al., 2012). If Prox1 is missing post-mitotically in immature dentate granule cells, they differentiate into neurons with the characteristic morphology and gene expression pattern of CA3 pyramidal cells (Iwano et al., 2012). In the absence of Prox1, intermediate progenitor cells are reduced and dentate granule cells cannot terminally differentiate (Lavado et al., 2010). As revealed by Fuzik et al. (2019), excitatory IG neurons lack Prox1 mRNA. We expanded this finding by studying the protein expression of Prox1 in the IG and FC during postnatal development. Our results revealed that IG pyramidal cells do not express Prox1 at any postnatal stage, precluding the same fate of dentate granule cells and IG neurons.

To corroborate our interpretation that IG neurons have a different developmental fate than dentate granule cells, we took advantage of a transgenic mouse model expressing eGFP under the transcriptional control of POMC genomic sequences. eGFP expression is also seen in adult-born dentate granule cells that are generated by adult stem cells. Therefore, absence of eGFP expression in the IG and FC in this transgenic mouse model excludes that these neurons have the developmental fate of dentate granule cells, despite their dentate granule cell–like morphology.

### The IG as a Distinct Subregion of the Hippocampal Formation

There has been an ongoing debate on the classification of the IG, as morphological studies so far did not allow a clear assignment to any distinct brain structure. Künzle (2004) divided the IG of the hedgehoc tenrec into an anterior precallosal, dorsal supracallosal, and posterior postcallosal portion. This subdivision is also confirmed in our study in the mouse IG. While the term *indusium griseum* is generally used in the neuroanatomical nomenclature to designate all three parts together (Paxinos and Watson, 2009), the precallosal part was also considered separately and referred to as the so-called anterior hippocampal continuation (Wyss and Sripanidkulchai, 1983). The latter term has also been used to combine the IG and the tenia tecta (Crosby and Schnitzlein, 1982) as part of the olfactory cortex as both receive input from the main olfactory bulb (Wyss and Sripanidkulchai, 1983). In the present study, we confirmed that characteristic histoarchitectonic features are common to the different parts of the IG along its anterior–posterior extent. Moreover, we emphasize that, based on our findings, a continuum between IG and DG can be excluded. Instead, the IG adjoins the hippocampal CA2 region via the FC. However, our study emphasizes that the FC is not identical with the medial CA2 region, as earlier stated (Laeremans et al., 2013). FC and medial CA2 can be distinguished from each other by the perpendicular orientation of their principal cell layers and by differences concerning their developmental protein expression patterns, in particular PCP4 expression. Taking into account our above described experimental findings, we support the interpretation of the IG as an own distinct subregion of the hippocampal formation, in line with a previously proposed definition (Künzle, 2004).

### Toward Exploring Functions of the IG

What could be the functional role of the IG? Although the hippocampal formation is best known for its role in learning and memory and loss of memory in the aging brain (Fjell et al., 2014), in spatial representation and navigating cognition (Bellmund et al., 2018), more recent studies also underpin its involvement in modulating social memory, and particular the CA2 region has been shown to play a crucial role in behavior (Hitti and Siegelbaum, 2014; Dudek et al., 2016; Montagrin et al., 2018; Oliva et al., 2020). While our present study clearly precludes dentate granule cell identity of IG neurons as earlier proposed, we found in contrast that IG neurons express the CA2 markers Necab2 and PCP4. Along this line, IG neurons also express Amigo2 mRNA, which is otherwise specific for CA2 neurons of the hippocampus proper but not expressed in CA1 or CA3 (© 2015 Allen Institute for Brain Science. Allen Brain Atlas API. Available from: https://mouse.brain-map.org/experiment/thumbnails/71250310?image_type=ish&popup=true; Laeremans et al., 2013; Hitti and Siegelbaum, 2014). Thus, a potential role of the IG in social memory or in other functions related to behavior could be experimentally tested in a similar way as it has been elegantly performed earlier to explore the role of the CA2 region in social memory, i.e., by local inactivation of Amigo2-expressing neurons in the CA2 region of Amigo2-Cre mice (Hitti and Siegelbaum, 2014). Next, *ex vivo* IG explants were recently successfully used for pharmacological treatment of IG principal neurons (Fuzik et al., 2019). Similarly, the use of slice cultures, a well-established *in vitro* model for hippocampal development and function, may be straightforward to study development, function, and aging of IG neurons *in vitro*. In particular, IG slice cultures may in the future serve as a novel *in vitro* model to explore mechanisms that underlie the low vulnerability of IG neurons, notably secretagogin- and PCP4-expressing neurons, in neurodegeneration (Lippa et al., 1990; Lippa and Smith, 1992; Attems et al., 2008). All in all, further investigations on the IG and FC are needed to elucidate specific functions of these distinct hippocampal subregions.

## Data Availability Statement

All datasets generated for this study are included in the article/Supplementary Material, further inquiries can be directed to the corresponding authors.

## Ethics Statement

The animal study was reviewed and approved by the Landesamt für Natur, Umwelt und Verbraucherschutz Nordrhein-Westfalen, Postfach 101052, 45610 Recklinghausen, Germany.

## Author Contributions

MS, MvD, and EF designed the study and revised the manuscript. MS and H-WH conducted the study and collected the data. MS, MvD, EP-P, and EF analyzed the data, drafted the manuscript, and interpreted the data. All the authors have read and approved the final version of the submitted manuscript.

## Conflict of Interest

The authors declare that the research was conducted in the absence of any commercial or financial relationships that could be construed as a potential conflict of interest.
