# Corrigendum: Postnatal Developmental Expression Profile Classifies the Indusium Griseum as a Distinct Subfield of the Hippocampal Formation

**DOI:** 10.3389/fcell.2022.856519

**Published:** 2022-03-02

**Authors:** Marie Sanders, Elisabeth Petrasch-Parwez, Hans-Werner Habbes, Monika v. Düring, Eckart Förster

**Affiliations:** Department of Neuroanatomy and Molecular Brain Research, Ruhr-University Bochum, Bochum, Germany

**Keywords:** indusium griseum, fasciola cinerea, dentate gyrus, CA2 region, Necab2, Prox1, secretagogin, PCP4

In the original article, Supplementary Figure S1 was erroneously not included in the publication. In turn, parts of the caption of this figure were mistakenly embedded in the main text during the editing process. The missing Supplementary Figure S1 appears in Supplementary Material.

A correction has been made to **Results**, *Calbindin Expression Decreases During Postnatal IG and FC Development*

The original article text appeared as below:

“Beaded axons of calbindin-positive cells projected to the adjacent cortical areas, the corpus callosum, and to the contralateral hemisphere (Commissural fibers of the indusium griseum (IG). Coronal sections of the IG in the mouse brain showing fibers projecting to the contralateral IG. Calbindin immunostaining at p15 (A). Calbindin-immunostaining shows several beaded axons (arrowheads in A) crossing to the contralateral side. Scale bar for (A) = 50 μm). As already detected in the IG, the FC also contained a large number of calbindin-immunopositive neurons at p0 (**Figure 2C**), which subsequently decreased during postnatal development (**Figures 2G,K,O,S**).”

The corrected text appears below:

“Beaded axons of calbindin-positive cells projected to the adjacent cortical areas, the corpus callosum, and to the contralateral hemisphere (Supplementary Figure S1A). As already detected in the IG, the FC also contained a large number of calbindin-immunopositive neurons at p0 (**Figure 2C**), which subsequently decreased during postnatal development (**Figures 2G,K,O,S**).”

Another correction has been made to **Results**, *Secretagogin Emerges Late During Postnatal IG and FC Development*

The original article text appeared as below:

“The majority of secretagogin-positive dendrites were oriented toward the molecular layer; some dendrites traversed to the contralateral IG (Commissural fibers of the indusium griseum (IG). Coronal sections of the IG in the mouse brain showing fibers projecting to the contralateral IG. Secretagogin immunostaining at 6 months of age (B). Secretagogin immunostained dendrites (arrowheads in B) crossing to the contralateral side. Scale bar for (A,B) in (B) = 50 μm). The heterogeneity, laminar distribution, and developmental expression pattern of secretagogin-positive cells in the FC were similar to those of the anterior IG (**Figures 3C,G,K**).”

The corrected text appears below:

“The majority of secretagogin-positive dendrites were oriented towards the molecular layer; some dendrites traversed to the contralateral IG (Supplementary Figure S1B). The heterogeneity, laminar distribution, and developmental expression pattern of secretagogin-positive cells in the FC were similar to those of the anterior IG (**Figures 3C,G,K**).”

The authors apologize for these errors and state that this does not change the scientific conclusions of the article in any way. The original article has been updated.

## Supplementary Material

Supplementary Figure S1 Commissural connections of the indusium griseum (IG). Coronal sections of the IG in the mouse brain showing fibers projecting to the contralateral IG. Calbindin immunostaining at p15 **(A)** and secretagogin immunostaining at 6 months of age **(B)**. Calbindin immunostaining shows several beaded axons (arrowheads in A) and secretagogin immunostaining shows dendrites (arrowheads in B) crossing to the contralateral hemisphere. Scale bar for **(A,B)** in **(B)** = 50 μm.

