# Supplementary material for: Corrigendum: Postnatal Developmental Expression Profile Classifies the Indusium Griseum as a Distinct Subfield of the Hippocampal Formation
Source: Front Cell Dev Biol. 2022 Mar 2;10:856519. doi: 10.3389/fcell.2022.856519 (PMC8924673; doi:10.3389/fcell.2022.856519)
Supplement: Supplementary file 1 [file DataSheet2.docx]

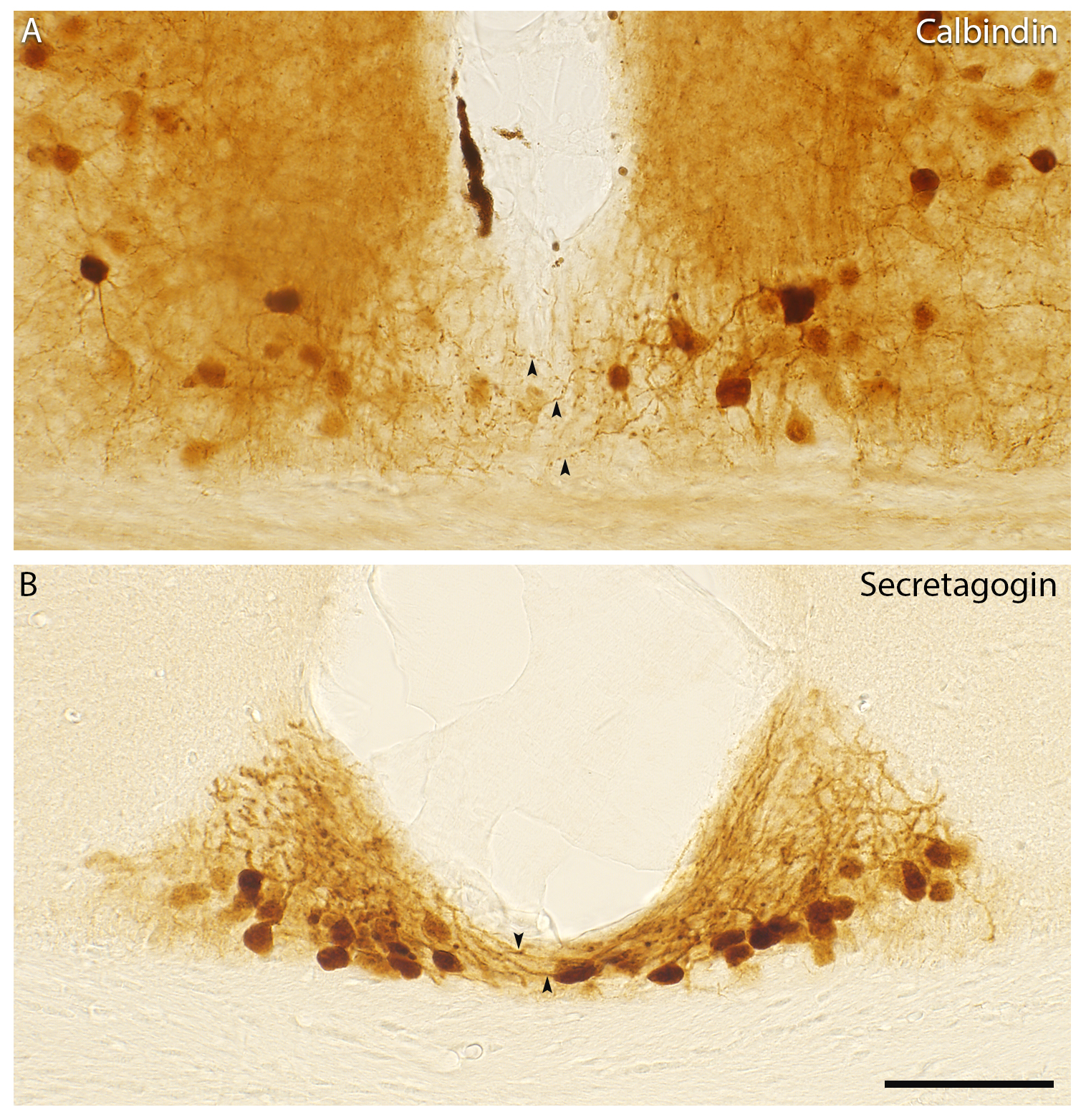


Supplementary Figure S1

Commissural connections of the indusium griseum (IG)

Coronal sections of the IG in the mouse brain showing fibers projecting to the contralateral IG. Calbindin immunostaining at p15 (A) and secretagogin immunostaining at 6 months of age (B). Calbindin immunostaining shows several beaded axons (arrowheads in A) and secretagogin immunostaining shows dendrites (arrowheads in B) crossing to the contralateral hemisphere.

Scale bar for A and B in B=50µm.
